# Enhancing Patient Care Through Improved Escalation Planning and Documentation: A Quality Improvement Project at a District General Hospital

**DOI:** 10.7759/cureus.86074

**Published:** 2025-06-15

**Authors:** Rana Maqsood, Mudasar Aziz, Katherine Hartley, Taimoor Hassan, Sijjad Ijaz, Haasin Ahmad, Abu Ajela Sreh

**Affiliations:** 1 Internal Medicine, Doncaster & Bassetlaw Teaching Hospitals NHS Foundation Trust, Doncaster, GBR; 2 Stroke, Doncaster Royal Infirmary, Doncaster, GBR; 3 Gastroenterology and Hepatology, Sheffield Teaching Hospital, Sheffield, GBR; 4 Emergency Medicine, Sheffield Teaching Hospital, Sheffield, GBR; 5 Gastroenterology, Sheffield Teaching Hospital, Sheffield, GBR

**Keywords:** clinical audit system, gastroenterology, nhs guidelines, nice guidelines, quality assessment in healthcare

## Abstract

Introduction

Clear escalation plans are critical to patient safety, particularly during periods of out-of-hours work. This is supported by guidance developed by the Royal College of Physicians, which states that all patients should have an escalation plan documented. Despite this, documentation is often inconsistent due to the complexity and time-consuming nature of these decisions. Nevertheless, accessibility to this information is imperative for effective handover between clinical teams and contributes towards delivering safe patient care.

Methods

The Plan-Do-Study-Act (PDSA) cycle methodology was used. Thirty patient notes were reviewed to assess the clarity of documentation regarding do not attempt cardiopulmonary resuscitation (DNAR) decisions, escalation planning, and weekend handover. Based on these findings, a standardised proforma was developed to evaluate the impact of the intervention on clinical practice. Subsequent PDSA cycles were implemented across the Gastroenterology ward.

Results

Following the introduction of the proforma, there were significant improvements in the quality of documentation. DNAR documentation increased from 47% to 90%, escalation planning improved from 23% to 81%, and weekend handover documentation rose from 47% to 100%.

Conclusion

The use of a standardised proforma improved the documentation and handover of patients in the Gastroenterology ward. This project demonstrated the positive impact of using a structured format to record key clinical information, thereby contributing to safer patient care. As a result, the proforma has been adopted by other wards within the hospital.

## Introduction

A critical aspect of patient care is escalation planning, particularly during the out-of-hours periods when resources in healthcare can be scarce and clinicians can be caring for larger numbers of patients whom they are unfamiliar with. Previous studies have demonstrated that weekend inpatients in UK hospitals have a higher risk of adverse events compared to those admitted during the working week [1,2].

Deciding escalation plans requires a holistic approach to the individual and a good understanding of their medical history, along with discussion with the patient and their family. This determines a “ceiling of care,” which may range from intensive care support to ward-based care, along with considering do not attempt cardiopulmonary resuscitation (DNAR) status. When escalation plans are decided and clearly documented, this facilitates optimal care to be delivered out-of-hours, in addition to reducing stress among clinicians [3,4].

The handover of patient care has been identified as a point at which errors can occur, which can have a deleterious effect on patient safety [5]. The Royal College of Physicians states that an escalation plan for all patients should be in place before the weekend, as part of comprehensive planning for patient care, considering their current condition and anticipating deteriorations [6]. Effective handovers are an integral aspect of patient safety, ensuring clinicians have the pertinent information necessary to care for patients effectively [7].

In order to address this, an audit was conducted at a district general hospital (DGH) in the North of England, which has approximately 500 inpatient beds, to assess the documentation of escalation plans and DNAR status for patients in the Gastroenterology ward, with the aim of improving the quality and safety of care delivered.

The aim of this project was to have the escalation status documented after three months for 80% of all patients in the gastroenterology ward. The project focused on evaluating and improving the documentation of the following three key components essential to safe patient care during out-of-hours periods: escalation plans, DNAR decisions, and weekend handover summaries.

## Materials and methods

Baseline assessment

The project utilised the Plan-Do-Study-Act (PDSA) format. This involved reviewing 30 patient notes at the end of each working week to determine if critical information was documented. This sample included all patients in the gastroenterology ward. The ward was selected for its manageable size and consistency of patients, making it a feasible site to conduct a pilot intervention. Data were collected with regards to clarity of escalation planning, documentation of the DNAR status, patient demographics, and a summary of the patient’s management. This approach ensured complete sampling of the ward population at the time of review, avoiding selection bias. Multiple team members were involved in data collection, and to ensure inter-rater reliability, all contributors received a joint training session beforehand. The same group of doctors on the rotation carried out data collection across all PDSA cycles to maintain consistency. This included discussion and clarification of what constituted adequate documentation in each domain.

Development of a standardised proforma

**Figure 1:**
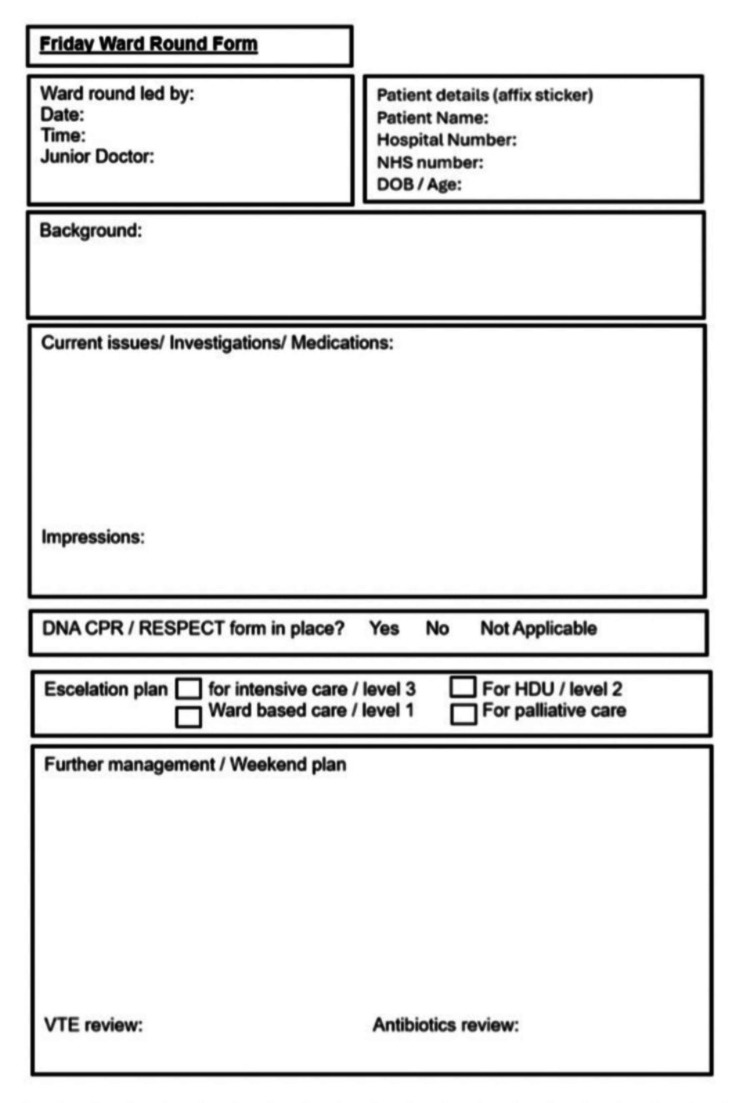
Weekend escalation planning and handover proforma. A standardized form designed to support documentation of DNAR status, escalation planning, and a concise summary of patient management for weekend ward rounds. DNAR, do not attempt cardiopulmonary resuscitation

Following the analysis of the initial data, a standardised proforma was developed (Figure 1). This proforma included a space for DNAR documentation, escalation planning, and even a weekend handover statement. The goal was to create a comprehensive, concise, and clear summary of each patient case. This was presented to the gastroenterology multidisciplinary team and governance meetings to ascertain feedback before being implemented in clinical practice. Modifications were made accordingly, for example, incorporating venous thromboembolism (VTE) prophylaxis and a dedicated weekend plan section.

Implementation and review

The proforma was circulated to all the consultants and subsequently modified in relation to their feedback. This allowed for changes to be made, such as the inclusion of the weekend plan and VTE prophylaxis. The proforma was printed on pink paper and distributed in the ward to use in place of the traditional continuation sheets. Pink paper was selected as this made the document easily identifiable in the patients’ notes and the colour was not being used for other documents.

The implementation of the proforma was communicated to all on-call staff across all divisions via the hospital’s weekly communications email. Two further PDSA cycles followed, which aimed at assessing the impact of the proforma on documentation. To assess sustainability and ensure adherence to the intervention, usage was monitored monthly, and plans were made to integrate the proforma into the induction process for new clinical teams rotating onto the ward.

## Results

On introduction of the proforma, the documentation practices in the Gastroenterology ward improved. Details of PDSA cycles are summarised in Table 1.

**Table 1:** Results Documentation rates across PDSA cycles 1–4 for DNAR decisions, escalation plans, and weekend handover statements. DNAR, do not attempt cardiopulmonary resuscitation; PDSA, Plan-Do-Study-Act

	PDSA 1	PDSA 2	PDSA 3	PDSA 4
DNAR decision (%)	47%	36%	84%	90%
Escalation plan (%)	23%	50%	68%	81%
Weekend handover (%)	47%	54%	96%	100%

DNAR documentation

Documentation of the DNAR status increased from 47% to 90% of case notes. This highlights the effectiveness of the proforma in ensuring vital information is clearly documented. This is an increase of 91%.

Escalation planning

Escalation documentation improved from 23% to 81%, an increase of 252%. The proforma provided a way of documenting clinically significant plans, ensuring that this information is clear and accessible to clinicians.

Weekend handover

The documentation of weekend handovers increased by 112%, from 47% to 100%. This facilitates effective handover, which is a vital step in maintaining patient safety as it helps to avoid miscommunication and errors.

## Discussion

Admissions to a hospital on a weekend have been shown to be associated with an increase in 30-day mortality compared to admissions which take place during standard working hours [2]. A significant factor contributing towards safe patient care is the quality and effectiveness of handover.

Studies have shown that handover can detrimentally affect patient safety because it is vulnerable to error given that it is an aspect of clinical work where there can be significant variability in the detail and accuracy [8,9]. This highlights handover as a source of preventing patient harm as the process can be impacted by human and systematic errors [10].

The Royal College of Physicians and Royal College of Surgeons have produced guidelines recommending that all patients should have a resuscitation status documented in written handovers; nevertheless, in clinical practice, one or more vital aspects are frequently omitted [11-14]. Electronic handovers for inpatients at the weekend have been shown to improve patient safety, reduce medical errors, and enhance continuity of care [15].

A study by Bhabra et al. [16] found that over a typical weekend, there can be up to five handovers, where only 2.5% of information from the first handover is retained to the final handover when there is no written record. If notes are taken, 85.5% of information is retained, and this rises to 99% if a proforma is used.

This project demonstrates the benefits of utilising a standardised proforma to enhance the quality of handovers which can benefit patient care and safety. The use of this documentation ensures that critical patient information was handed over whilst reducing the risk of error. Recording this information improves consistency, and this information is accessible to the out-of-hours team facilitating the delivery of optimal patient care during these periods.

Sustainability and future directions

The sustainability of the changes implemented was reassessed with a further PDSA cycle. As a result of the benefits of this project, the standardised proforma was presented at the governance meeting and subsequently approved for use in other medical wards.

The following points are recommended in order to maintain and enhance the quality of documentation in relation to patient care. Ongoing training of the medical and nursing staff is required to familiarise the staff with the proforma and provide updates at regular intervals. This proforma can be extended to other departments in the hospital to achieve a standardised approach to documentation throughout the trust. A feedback process should be established to enable staff to comment on the proforma’s use, especially as it is introduced in new departments. This will help guide future improvements and ensure that the template remains beneficial for clinicians and patient handovers. Further periodic reevaluations of compliance are required to ensure that documentation standards are maintained and to inform further interventions. Further work should also seek to involve patients and their families in early planning for escalation as this enables the development of patient-centred care and goal-directed plans.

Limitations

Several limitations can be identified from this audit. It reviewed data from only 30 sets of notes at a single district general hospital ward, making the findings vulnerable to both small-sample imprecision and site-specific cultural or staffing factors, which limits external validity. Moreover, the outcomes measured were process metrics, such as completion rates for DNAR status, escalation plans, and handover proformas, rather than clinical endpoints, such as adverse events, length of stay, or mortality. This means that the studies demonstrate improved documentation but cannot confirm enhanced patient safety. Finally, as successive PDSA cycles were carried out over a short time frame and staff were aware of ongoing observation, Hawthorne effects and regression to the mean may have exaggerated the apparent gains. The brief follow-up period also offers limited insight into whether behaviour changes are sustained once the project concludes. Furthermore, the use of a paper-based proforma raises questions about scalability in hospitals increasingly moving towards electronic handover systems. Future iterations of this intervention may need to explore integration with electronic platforms to ensure a wider adoption and long-term sustainability.

## Conclusions

It has been proved that the introduction of a standardised proforma at this hospital enhanced the documentation and handover process in the Gastroenterology ward. By utilising the PDSA methodology, improvements in documentation of DNAR status, escalation planning, and weekend handover were demonstrated. These findings are in accordance with the increasing body of evidence that clearly supports the use of standardised templates in order to improve patient handover, thus contributing towards patient safety and quality of care, particularly during periods of out-of-hours work.

The success of this intervention highlighted the value of structured documentation in clinical practice and created a useful model for other departments to use. By adopting this process, healthcare providers can improve the quality and consistency of vital patient handover, which can enhance patient care and safety.

## References

[1] Wise J (2012). New evidence of worse outcomes for weekend patients reignites call for seven day hospital services. BMJ.

[2] Freemantle N, Richardson M, Wood J (2012). Weekend hospitalization and additional risk of death: an analysis of inpatient data. J R Soc Med.

[3] Dahill M, Powter L, Garland L, Mallett M, Nolan J (2013). Improving documentation of treatment escalation decisions in acute care. BMJ Qual Improv Rep.

[4] Grainge C, Traer E, Fulton J (2005). Do weekend plan standard forms improve communication and influence quality of patient care?. Postgrad Med J.

[5] (2025). British Medical Association. Safe handover: safe patients. Guidance for clinical handover for clinicians and managers. https://www.rcseng.ac.uk/-/media/files/rcs/library-and-publications/non-journal-publications/safe-handovers.pdf.

[6] Zarkali A, Black D, Smee E, Deshraj A, Smallwood N (2014). Planning ahead: Improving escalation plans before the weekend. BMJ Qual Improv Rep.

[7] (2025). Emergency and acute medical care in over 16s (QS174). https://www.nice.org.uk/guidance/qs174.

[8] Bomba DT, Prakash R (2005). A description of handover processes in an Australian public hospital. Aust Health Rev.

[9] Thompson JE, Collett LW, Langbart MJ (2011). Using the ISBAR handover tool in junior medical officer handover: a study in an Australian tertiary hospital. Postgrad Med J.

[10] Royal College of Physicians (2011). Acute Care Toolkit 1: Handover. https://www.rcp.ac.uk/media/5q5hqwbx/acute-care-toolkit-1-handover.pdf.

[11] Metz D, Chard D, Rhodes J, Pounder P (2004). Continuity of Care for Medical Inpatients: Standards of Good Practice. https://books.google.co.uk/books?id=dWhRxmnZ7Z8C&printsec=frontcover&source=gbs_ge_summary_r&cad=0#v=onepage&q&f=false.

[12] The Royal College of Surgeons of England (2007). Safe Handover: Guidance From the Working Time Directive Working Party. RCS.

[13] Arora V, Johnson J, Lovinger D, Humphrey HJ, Meltzer DO (2005). Communication failures in patient sign-out and suggestions for improvement: a critical incident analysis. Qual Saf Health Care.

[14] Pfeffer PE, Nazareth D, Main N, Hardoon S, Choudhury AB (2011). Are weekend handovers of adequate quality for the on-call general medical team?. Clin Med (Lond).

[15] Govier M, Medcalf P (2012). Living for the weekend: electronic documentation improves patient handover. Clin Med (Lond).

[16] Bhabra G, Mackeith S, Monteiro P, Pothier DD (2007). An experimental comparison of handover methods. Ann R Coll Surg Engl.

